# Clinical Outcomes of Double Half‐Pigtail Versus Straight Plastic Stents for Endoscopic Biliary Drainage of Malignant Distal Biliary Obstruction

**DOI:** 10.1002/deo2.70446

**Published:** 2026-09-29

**Authors:** Wataru Yoshioka, Mutsumi Tasaki, Hinata Tasaki, Hikaru Fujimoto, Shuzo Yunotani, Chika Inadomi, Shotaro Noge, Yoshihito Kubotsu, Futa Koga

**Affiliations:** ^1^ Department of Hepatobiliary and Pancreatology Saga Medical Center Koseikan Saga Japan

**Keywords:** endoscopic retrograde cholangiopancreatography, malignant distal biliary obstruction, plastic stent, recurrent biliary obstruction, stent migration

## Abstract

**Objectives:**

The clinical performance of double half‐pigtail plastic stents (DHPs) for malignant distal biliary obstruction (MDBO) remains unclear. We compared DHPs with straight plastic stents (STs) regarding the timing, incidence, and causes of recurrent biliary obstruction (RBO).

**Methods:**

This single‐center retrospective cohort study evaluated endoscopic retrograde cholangiopancreatography‐guided transpapillary single plastic stent placements for MDBO performed between October 2022 and March 2026. For the primary analysis, only initial stenting episodes were included. The primary endpoint was the time to RBO (TRBO), which was analyzed using Kaplan–Meier and log‐rank methods.

**Results:**

A total of 131 stent‐placement episodes in 86 patients were eligible. The primary analysis included 72 patients undergoing initial stenting, comprising 20 patients in the DHP group and 52 patients in the ST group. The DHP and ST groups had a similar median TRBO (116 vs. 119 days; log‐rank *p* = 0.726), incidence of RBO (40.0% vs. 46.2%; *p* = 0.638), and incidence of stent occlusion (40.0% vs. 32.7%; *p* = 0.560). No migration occurred with DHPs, whereas seven migration events occurred with STs (0% vs. 13.5%; *p* = 0.084). Both groups had similar adverse event rates (0% vs. 11.5%; *p* = 0.113).

**Conclusions:**

DHPs did not prolong the TRBO or reduce its incidence compared with STs. However, migration‐related RBO occurred only with STs. The potential advantage of DHPs may be a lower incidence of stent migration rather than longer overall stent patency.

**Trial Registration:**

N/A

## 1 Introduction

The standard approach for managing malignant distal biliary obstruction (MDBO) is endoscopic retrograde cholangiopancreatography (ERCP)‐guided transpapillary biliary drainage [1]. Self‐expandable metal stents generally provide longer stent patency than plastic stents and are recommended for patients requiring preoperative or palliative biliary drainage [2, 3]. However, plastic stents remain clinically useful in selected situations, such as when short‐term drainage is intended, when the diagnosis or treatment strategy has not yet been finalized, or when easy stent removability and exchangeability are important [4]. Therefore, optimizing the performance of plastic stents remains a relevant issue in daily clinical practice.

A major limitation of transpapillary biliary stenting is recurrent biliary obstruction (RBO). The Tokyo Criteria 2014 defines RBO as a composite endpoint that includes both stent occlusion and stent migration and recommends the use of the time to RBO (TRBO) as a standardized outcome for evaluating stent patency [5]. The occlusion of plastic stents is associated with bacterial biofilm formation and the accumulation of biliary sludge, debris, and food components [6]. In addition, stent migration can compromise bile drainage, which results in RBO and the need for biliary reintervention [7]. Although straight plastic stents (STs) are widely used because of their simplicity and availability, migration may occur despite their anchoring flaps [8].

To address these issues, double half‐pigtail plastic stents (DHPs) with a preloaded system and anti‐migration design (Through & Pass Double Pit; Gadelius Medical K.K.) were developed (Figure 1). DHPs are preloaded onto the delivery catheter using a threaded connection and have multiple side holes at both ends. Unlike conventional fully curved pigtail stents, DHPs have a semicircular curved configuration, which is expected to reduce the risk of stent implantation into the liver parenchyma. A recent retrospective study of patients with acute calculous cholangitis showed that DHPs are associated with lower rates of stent occlusion, stent migration, and RBO, as well as a longer TRBO, compared with STs [9]. However, findings from cases of bile duct stone disease may not be directly applicable to MDBO because malignant biliary strictures may themselves influence stent migration by acting as an anchoring point [7, 8]. It remains unclear whether DHPs prolong the TRBO or reduce migration‐related RBO compared with STs in patients with MDBO. Therefore, we conducted a comparative evaluation of DHPs and STs for endoscopic transpapillary biliary drainage in patients with MDBO.

**FIGURE 1:**
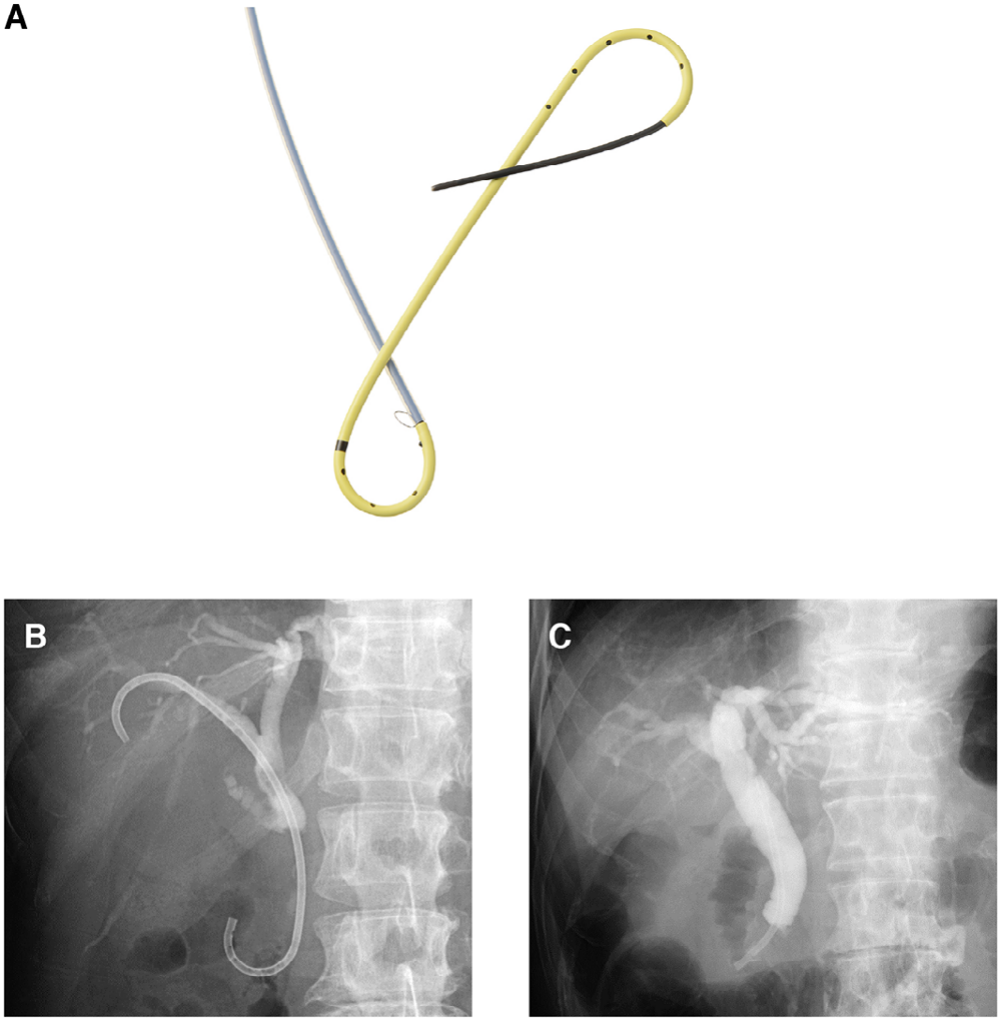
Representative images of double half‐pigtail and straight plastic stents. (A) Photograph of a double half‐pigtail plastic stent (Through & Pass Double Pit; Gadelius Medical K.K., Tokyo, Japan). (B) Fluoroscopic image of the transpapillary placement of a double half‐pigtail plastic stent for malignant distal biliary obstruction. The proximal end of the stent is positioned in the hepatic duct or intrahepatic bile duct, and the distal end is positioned in the duodenum. (C) Fluoroscopic image of the transpapillary placement of a straight plastic stent for malignant distal biliary obstruction.

## 2 Methods

### 2.1 Study Design and Patients

This single‐center, retrospective cohort study reviewed eligible stent‐placement episodes in patients with MDBO who underwent successful ERCP‐guided transpapillary placement of a single plastic stent between October 2022, when DHPs were introduced at our institution, and March 2026.

Stent‐placement episodes were eligible if a conventional side‐viewing duodenoscope was used to successfully place a single plastic stent across a malignant distal biliary stricture. The study included both initial stenting episodes and subsequent stenting episodes. Initial stenting was defined as the first endoscopic transpapillary biliary stent placement for MDBO. Episodes involving metal stent placement, multiple plastic stent placements, endoscopic ultrasound‐guided biliary drainage, percutaneous transhepatic biliary drainage, hilar biliary obstruction, benign biliary stricture, surgically altered anatomy requiring balloon‐assisted enteroscopy, technical failure of transpapillary stent placement, or insufficient follow‐up information were excluded. Disease status and Eastern Cooperative Oncology Group (ECOG) performance status were assessed, and ongoing systemic chemotherapy was recorded at the time of the index ERCP. For the primary analysis, only initial stenting episodes were included. Patients whose first eligible study episode was non‐initial stenting were excluded from the primary analysis. Patients in the primary analysis were divided into the DHP group and the ST group according to the stent type used for initial stenting.

### 2.2 Endoscopic Procedures

All procedures were performed by experienced endoscopists using a conventional side‐viewing duodenoscope and standard ERCP techniques. After biliary cannulation, cholangiography was performed to assess the biliary stricture and bile duct configuration. Endoscopic sphincterotomy was performed at the discretion of the endoscopist.

The DHP used in this study was the Through & Pass Double Pit stent (Gadelius Medical K.K., Tokyo, Japan). The STs used in this study were the Flexima Biliary Stent System (Boston Scientific Japan K.K., Tokyo, Japan), QuickPlace V biliary stent (Olympus Medical Systems Corp., Tokyo, Japan), REGULUS Biliary Tube Stent System (Japan Lifeline Co., Ltd., Tokyo, Japan), and Oasis One Action stent introduction system preloaded with an ST‐2 Soehendra Tannenbaum biliary stent (Cook Medical Japan G.K., Tokyo, Japan).

There was no predefined institutional protocol for selecting the stent type or diameter. The type and diameter of the plastic stent were selected at the discretion of the endoscopist based on the stricture anatomy, bile duct configuration, and clinical condition of the patient. For subsequent stent placement, the clinical performance of the previously placed stent was also taken into consideration. The proximal end of the DHP was placed in the hepatic duct or intrahepatic bile duct to fit its curved configuration, whereas the position of the ST was determined in accordance with the biliary stricture and bile duct configuration. The location of the proximal end of the DHP was classified as left or right according to the hepatic duct in which it was positioned. The maximum diameter of the common bile duct proximal to the biliary stricture was measured on cholangiography.

### 2.3 Outcomes and Definitions

The primary endpoint was the TRBO. The secondary endpoints were the incidence of RBO, causes of RBO, and adverse events.

RBO was defined as biliary obstruction requiring biliary reintervention after plastic stent placement. The causes of RBO were classified as stent occlusion, proximal stent migration, and distal stent migration. Stent occlusion was defined as RBO without evidence of stent migration on imaging or endoscopic findings. Proximal stent migration was defined as inward migration of the stent toward the bile duct, whereas distal stent migration was defined as outward migration of the stent toward the duodenum or spontaneous stent dislodgement. Only migration events requiring biliary reintervention were counted as RBO events.

The TRBO was defined as the interval from plastic stent placement to biliary reintervention for RBO. Episodes without RBO were censored at the time of planned stent exchange, conversion to metal stent placement, surgery, death without RBO, or last available follow‐up.

Cholangitis at index ERCP was diagnosed in accordance with the Tokyo Guidelines 2018 [10]. Adverse events related to ERCP were evaluated in accordance with the Tokyo Criteria 2024 [11].

### 2.4 Sensitivity Analysis

To account for the potential influence of stent diameter, a sensitivity analysis limited to 8.5‐Fr stents was performed.

### 2.5 Statistical Analysis

Statistical analyses were performed using IBM SPSS Statistics, version 22.0 (IBM Corp., Armonk, NY, USA) and EZR (Jichi Medical University, Saitama, Japan), a graphical user interface for R (The R Foundation for Statistical Computing, Vienna, Austria) [12]. Continuous variables are presented as median and interquartile range and compared using the Mann–Whitney U test. Categorical variables are presented as numbers and percentages and compared using the *χ* ^2^ test. The TRBO was estimated using the Kaplan–Meier method and compared using the log‐rank test. TRBO in non‐initial stenting episodes was summarized descriptively because some patients contributed multiple episodes. Migration events were additionally summarized descriptively using all eligible stent‐placement episodes. For the 14 patients excluded from the primary analysis because their first eligible study episode was non‐initial stenting, the previously placed biliary device and reason for repeat ERCP were summarized descriptively. All statistical tests were two‐sided, and statistical significance was set at *p* < 0.05.

## 3 Results

### 3.1 Baseline and Procedural Characteristics

A total of 131 successful single plastic stent placement episodes in 86 patients were eligible. Fourteen patients whose first eligible study episode was non‐initial stenting were excluded from the primary analysis. The primary‐analysis cohort therefore comprised 72 patients undergoing initial stenting, including 20 patients in the DHP group and 52 patients in the ST group (Figure 2).

**FIGURE 2:**
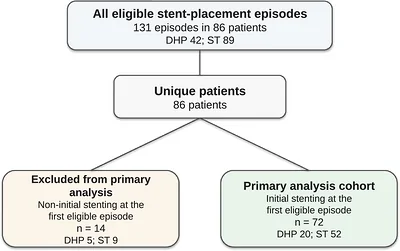
Study flow. A total of 131 eligible stent‐placement episodes were identified in 86 patients. For the primary analysis, 72 patients whose first eligible episode was initial stenting were included, whereas 14 patients whose first eligible episode was non‐initial stenting were excluded. DHP, double half‐pigtail plastic stent; ST, straight plastic stent.

The baseline and procedural characteristics are summarized in Table 1. There were no significant differences between the DHP group and the ST group in age, sex, primary disease, disease status, ECOG performance status, undergoing chemotherapy, cholangitis at index ERCP, inflammatory markers, serum total bilirubin concentration, endoscopic sphincterotomy, duodenal invasion, stricture length, or maximum common bile duct diameter. The stent diameter did not significantly differ between the two groups (*p* = 0.099). An 8.5‐Fr stent was used in 18 patients (90.0%) in the DHP group and 39 patients (75.0%) in the ST group, whereas stents with a diameter of 10 Fr or larger were used only in the ST group.

**TABLE 1:** Baseline and procedural characteristics in accordance with stent type.

Characteristic	DHP group (*n* = 20)	ST group (*n* = 52)	*p*‐value
Age (years)	79.0 (71.8–85.0)	75.5 (67.8–82.3)	0.263
Male sex	13 (65.0)	29 (55.8)	0.477
Primary disease			0.162
Pancreatic cancer	8 (40.0)	33 (63.5)	
Bile duct cancer	9 (45.0)	12 (23.1)	
Ampullary cancer	1 (5.0)	5 (9.6)	
Other	2 (10.0)	2 (3.8)	
Disease status			0.602
Resectable	6 (30.0)	19 (36.5)	
Unresectable or recurrent	14 (70.0)	33 (63.5)	
ECOG performance status			1.000
0–1	15 (75.0)	39 (75.0)	
≥2	5 (25.0)	13 (25.0)	
Undergoing chemotherapy	3 (15.0)	3 (5.8)	0.204
Cholangitis at index ERCP	4 (20.0)	8 (15.4)	0.638
WBC (/µL)	6000 (4275–7000)	6150 (5000–7525)	0.567
CRP (mg/dL)	0.78 (0.42–1.59)	1.19 (0.46–2.92)	0.493
Total bilirubin (mg/dL)	4.2 (2.5–8.6)	7.8 (3.2–13.3)	0.123
Endoscopic sphincterotomy	18 (90.0)	48 (92.3)	0.751
Duodenal invasion	2 (10.0)	4 (7.7)	0.751
Stricture length (mm)	15.5 (11.0–18.3)	16.5 (11.8–25.3)	0.701
Maximum common bile duct diameter (mm)	14.0 (12.8–16.0)	15.0 (13.0–18.0)	0.483
Stent diameter			0.099
7 Fr	2 (10.0)	3 (5.8)	
8.5 Fr	18 (90.0)	39 (75.0)	
≥10 Fr	0 (0.0)	10 (19.2)	

Data are presented as median (interquartile range) or *n* (%). *p*‐Values were calculated using the Mann–Whitney U test for continuous variables and Pearson's *χ* ^2^ test for categorical variables.

Abbreviations: CRP, C‐reactive protein concentration; DHP, double half‐pigtail plastic stent; ECOG, Eastern Cooperative Oncology Group; ERCP, endoscopic retrograde cholangiopancreatography; Fr, French; ST, straight plastic stent; WBC, white blood cell count.

Among the 14 patients excluded because of non‐initial stenting, the previously placed biliary devices were STs in 11 patients, double‐pigtail plastic stents in two, and a self‐expandable metal stent in one; further details are provided in Table S1.

### 3.2 Clinical Outcomes

The clinical outcomes in accordance with the stent type are shown in Table 2. The median follow‐up period was 42.5 days in the DHP group and 50.0 days in the ST group (*p* = 0.930). Both groups had similar incidences of RBO (*p* = 0.638) and stent occlusion (*p* = 0.560). Stent migration occurred only in the ST group, with seven migration events in the ST group and none in the DHP group (0% vs. 13.5%; *p* = 0.084). In the ST group, three migrations were proximal, and four were distal. Thus, all RBO events in the DHP group were caused by stent occlusion, whereas RBO events in the ST group were caused by both stent occlusion and stent migration.

**TABLE 2:** Clinical outcomes in accordance with stent type.

Outcome	DHP group (*n* = 20)	ST group (*n* = 52)	*p*‐value
Median follow‐up period (days)	42.5 (28.8–88.5)	50.0 (23.5–94.3)	0.930
Median TRBO (days)	116	119	0.726
Recurrent biliary obstruction	8 (40.0)	24 (46.2)	0.638
Stent occlusion	8 (40.0)	17 (32.7)	0.560
Stent migration	0 (0.0)	7 (13.5)	0.084
Proximal migration	0 (0.0)	3 (5.8)	0.273
Distal migration	0 (0.0)	4 (7.7)	0.202
Adverse events	0 (0.0)	6 (11.5)	0.113

Data are presented as median or *n* (%). The *p*‐value for the TRBO was calculated using the log‐rank test, the *p*‐value for the follow‐up period was calculated using the Mann–Whitney U test, and other *p*‐values were calculated using Pearson's *χ* ^2^ test. All adverse events were pancreatitis after endoscopic retrograde cholangiopancreatography.

Abbreviations: DHP, double half‐pigtail plastic stent; ST, straight plastic stent; TRBO, time to recurrent biliary obstruction.

The proximal end of the DHP was positioned in the left hepatic duct in 15 patients (75.0%) and in the right hepatic duct in 5 patients (25.0%). RBO occurred in 6/15 (40.0%) and 2/5 (40.0%), respectively, and no migration was observed in either subgroup. The TRBO did not significantly differ according to proximal stent location (log‐rank *p* = 0.877).

Among all 131 eligible stent‐placement episodes, 13 migration events occurred, all in the ST group, with a median time to migration of 58 days. Stent migration according to stent diameter is summarized in Table S2.

The adverse event rate was similar in both groups (*p* = 0.113). All adverse events were post‐ERCP pancreatitis. No bleeding, perforation, or procedure‐related death was observed in either group.

### 3.3 Time to RBO

There was no significant difference between the two groups in the TRBO (116 vs. 119 days; log‐rank *p* = 0.726; Table 2 and Figure 3).

**FIGURE 3:**
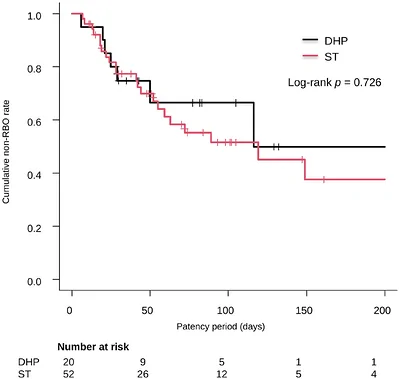
Kaplan–Meier curves for the time to recurrent biliary obstruction in accordance with stent type in the primary analysis cohort. Tick marks indicate censored observations, and the numbers at risk are shown below the plot. DHP, double half‐pigtail plastic stent; RBO, recurrent biliary obstruction; ST, straight plastic stent.

Among 59 non‐initial stenting episodes contributed by 37 patients, 22 involved DHPs and 37 involved STs. The median TRBO was 99 days in the DHP group and 125 days in the ST group. These results are presented descriptively because some patients contributed multiple episodes.

### 3.4 Sensitivity Analysis

In the 8.5‐Fr stent analysis, the TRBO did not significantly differ between the DHP and ST groups (222 vs. 214 days; log‐rank *p* = 0.962).

## 4 Discussion

In this single‐center retrospective cohort study, the median TRBO and overall RBO rate did not significantly differ between the DHP and ST groups. However, the causes of RBO differed: all RBO events in the DHP group were caused by stent occlusion, whereas stent migration occurred only in the ST group. These findings suggest that DHPs may reduce migration‐related RBO without prolonging the overall TRBO.

A recent retrospective study of patients with acute calculous cholangitis reported lower rates of stent occlusion, migration, and RBO and a longer TRBO with DHPs than with STs [9]. In contrast, we found that DHPs did not prolong the TRBO compared with STs in patients with MDBO. This discrepancy between studies may reflect the disease‐specific anatomy. The frequency and direction of plastic stent migration vary in accordance with the underlying disease, and malignant strictures may anchor stents [7, 8]. Thus, anti‐migration designs may confer different benefits in bile duct stone disease compared with MDBO.

The absence of migration in the DHP group was clinically notable but should not be attributed simply to the pigtail design. A study of patients with irretrievable bile duct stones reported that conventional double‐pigtail stents do not clearly reduce distal migration compared with STs [8]. However, in this previous study, the proximal tip was positioned above the stones, and the distal tip was positioned in the second portion of the duodenum, whereas conventional double‐pigtail stents were likely deployed within the extrahepatic bile duct [8, 9]. In contrast, the DHP used in our study had semicircular curves at both ends, and its proximal end was placed in the hepatic duct or intrahepatic bile duct. This configuration and placement may have contributed to the absence of migration, although the nonrandomized selection of stents makes this merely a hypothesis‐generating finding. RBO occurred in 40.0% of patients with both left‐ and right‐sided proximal placement of the DHP, and the TRBO did not significantly differ according to proximal stent location. However, the small number of patients in each subgroup precludes firm conclusions regarding the influence of proximal stent location on clinical outcomes.

Despite the absence of migration, DHPs did not prolong the TRBO. Although the incidence of stent occlusion was numerically higher in the DHP group, this finding should be interpreted cautiously. Stent occlusion and migration represented different modes of RBO, and the absence of migration in the DHP group may have shifted the observed mode of stent failure toward occlusion, whereas some stents in the ST group failed because of migration before occlusion could occur. Plastic stent occlusion is driven by bacterial biofilm formation and the accumulation of biliary sludge, debris, and food components [6]. In MDBO, stent dysfunction may also reflect malignant strictures, bile stasis, duodenobiliary reflux, infection, and the clinical course during anticancer treatment [6, 13]. Several factors, including stent diameter, configuration, and position, may have contributed to the numerically higher incidence of occlusion in the DHP group. Larger‐diameter stents were used only in the ST group, which may have contributed to the numerically lower occlusion rate in that group. In addition, the curved configuration and proximal placement of the DHP may have influenced bile flow or the accumulation of sludge and debris; however, these mechanisms could not be evaluated in the present study. Because of the limited number of occlusion events, the present study could not reliably determine factors associated with stent occlusion. Thus, the numerically higher occlusion rate does not establish that DHPs intrinsically increase the risk of stent occlusion. Therefore, reducing migration alone may not improve the overall TRBO; the potential benefit of DHPs may lie in their association with fewer migration‐related RBO events rather than longer stent patency.

Stent migration can cause RBO that requires biliary reintervention, and the retrieval of proximally migrated plastic stents can be technically difficult and occasionally unsuccessful [7, 14]. Accordingly, DHPs may represent a viable alternative plastic stent design in MDBO, particularly when avoidance of stent migration is considered clinically important, and the hepatic duct or intrahepatic bile duct can accommodate their curved configuration. Further studies are warranted to identify the patients most likely to benefit from DHP placement.

The present study has several limitations. First, its single‐center retrospective design and discretionary stent selection may have introduced selection bias and unmeasured confounding factors. Second, there were several stent types used in the ST group, and the effect of stent design could not be separated from that of stent placement position. Third, there was an imbalance in stent diameter between the groups. However, the 8.5‐Fr sensitivity analysis revealed findings that were consistent with the main analysis. Fourth, the small sample size of the DHP group limited the power to detect modest differences in the TRBO and uncommon adverse events. Finally, tumor progression and anticancer treatment during follow‐up were not evaluated.

In conclusion, DHPs did not significantly prolong the TRBO or reduce the overall RBO rate compared with STs in patients with MDBO. However, migration‐related RBO occurred only in the ST group. The potential advantage of DHPs may therefore be a lower incidence of migration‐related RBO rather than prolonged overall stent patency.

## Author Contributions

**Wataru Yoshioka**: conceptualization; methodology; investigation; data curation; formal analysis; visualization; project administration; writing – original draft; writing – review & editing. **Mutsumi Tasaki**: formal analysis; validation; writing – review & editing. **Hinata Tasaki**, **Hikaru Fujimoto**, **Shuzo Yunotani**, **Chika Inadomi**, **Shotaro Noge**, and **Yoshihito Kubotsu**: validation; writing – review & editing. **Futa Koga**: conceptualization; validation; writing – review & editing. All authors reviewed and approved the final version of the manuscript and agreed to be accountable for all aspects of the work.

## Funding

The authors have nothing to report.

## Ethics Statement

This study was conducted in accordance with the Declaration of Helsinki and was approved by the Institutional Review Board of Saga Medical Center Koseikan (approval number: 26‐07‐01‐01).

## Consent

The requirement for written informed consent was waived because of the retrospective nature of the study. This study used an opt‐out approach, and information about the study was made publicly available.

## Conflicts of Interest

The authors declare no conflicts of interest.

## Supporting information




**TABLE S1**: Previous biliary devices and reasons for repeat ERCP among the 14 non‐initial stenting episodes excluded from the primary analysis.

**TABLE S2**: Stent migration among all eligible stent‐placement episodes.

## Data Availability

The data that support the findings of this study are available from the corresponding author upon reasonable request.
