# Supplementary material for: Clinical Outcomes of Double Half‐Pigtail Versus Straight Plastic Stents for Endoscopic Biliary Drainage of Malignant Distal Biliary Obstruction
Source: DEN Open. 2026 Sep 29;7(1):e70446. doi: 10.1002/deo2.70446 (PMC13621336; doi:10.1002/deo2.70446)
Supplement: Supplementary file 1 [file DEO2-7-e70446-s001.docx]

**Table S2. Stent migration among all eligible stent-placement episodes**

| Characteristic | DHP group (n=42) | ST group (n=89) |
| --- | --- | --- |
| Stent migration | 0 (0.0) | 13 (14.6) |
| Median time to stent migration (days) | — | 58 (25–63) |
| Direction of migration |  |  |
| Proximal | 0 | 7 |
| Distal | 0 | 6 |
| Stent migration by stent diameter |  |  |
| 7 Fr | 0/4 (0.0) | 1/5 (20.0) |
| 8.5 Fr | 0/38 (0.0) | 12/62 (19.4) |
| ≥10 Fr | — | 0/22 (0.0) |

Migration data from all 131 eligible stent-placement episodes are presented descriptively. Of the 13 migration events, 7 were included in the primary analysis and 6 occurred in additional eligible episodes. DHP, double half-pigtail plastic stent; Fr, French; ST, straight plastic stent.
