# Supplementary material for: Clinical Outcomes of Double Half‐Pigtail Versus Straight Plastic Stents for Endoscopic Biliary Drainage of Malignant Distal Biliary Obstruction
Source: DEN Open. 2026 Sep 29;7(1):e70446. doi: 10.1002/deo2.70446 (PMC13621336; doi:10.1002/deo2.70446)
Supplement: Supplementary file 2 [file DEO2-7-e70446-s002.docx]

**Table S1. Previous biliary devices and reasons for repeat ERCP among the 14 non-initial stenting episodes excluded from the primary analysis**

| Characteristic | DHP group (n=5) | ST group (n=9) |
| --- | --- | --- |
| Previously placed biliary device |  |  |
| ST | 4 (80.0) | 7 (77.8) |
| Double-pigtail PS | 1 (20.0) | 1 (11.1) |
| SEMS | 0 (0.0) | 1 (11.1) |
| Reason for repeat ERCP |  |  |
| Planned exchange | 2 (40.0) | 7 (77.8) |
| Stent occlusion | 1 (20.0) | 1 (11.1) |
| Proximal migration | 1 (20.0) | 1 (11.1) |
| Distal migration | 1 (20.0) | 0 (0.0) |

Data are presented as n (%). Percentages are calculated within each current stent group. DHP, double half-pigtail plastic stent; ERCP, endoscopic retrograde cholangiopancreatography; PS, plastic stent; SEMS, self-expandable metal stent; ST, straight plastic stent.
